# Abdominal ultrasound stimulation alleviates DSS-induced colitis and behavioral disorders in mice by mediating the microbiota–gut–brain axis balance

**DOI:** 10.1016/j.neurot.2024.e00494

**Published:** 2024-11-22

**Authors:** Cong-Yong Gao, Yi-Ju Pan, Wei-Shen Su, Chun-Yi Wu, Ting-Yu Chang, Feng-Yi Yang

**Affiliations:** a Department of Biomedical Imaging and Radiological Sciences, National Yang Ming Chiao Tung University, Taipei, Taiwan; b Department of Psychiatry, Far Eastern Memorial Hospital, New Taipei City, Taiwan; c Department of Chemical Engineering and Materials Science, Yuan Ze University, Taoyuan City, Taiwan

**Keywords:** Abdominal ultrasound, IBD, Intestinal inflammation, Neuroinflammation, Microbiota

## Abstract

Inflammatory bowel disease (IBD) has the potential to induce neuroinflammation, which may increase the risk of developing neurodegenerative disorders. Ultrasound stimulation to the abdomen is a potential treatment for dextran sulfate sodium (DSS)-induced acute colitis. The present study aimed to investigate whether abdominal low-intensity pulsed ultrasound (LIPUS) can alleviate DSS-induced neuroinflammation through the microbiota–gut–brain axis. Male mice were fed DSS to induce ulcerative colitis. LIPUS stimulation was then applied to the abdomen at intensities of 0.5 and 1.0 ​W/cm^2^. Mouse biological samples were analyzed, and behavior was evaluated. [^18^F]FEPPA PET/CT imaging was employed to track and quantify inflammation in the abdomen and brain. Changes in the gut microbiota composition were analyzed using 16S rRNA sequencing. Abdominal LIPUS significantly inhibited the DSS-induced inflammatory response, repaired destroyed crypts, and partially preserved the epithelial barrier. [^18^F]FEPPA accumulation in the colitis-induced neuroinflammation in the abdomen and specific brain regions significantly decreased after LIPUS treatment. LIPUS maintained intestinal integrity by increasing zonula occludens and occludin levels, reduced lipopolysaccharide-binding protein and lipopolysaccharide levels in the serum, and improved behavioral dysfunctions. Moreover, LIPUS, at an intensity of 0.5 ​W/cm^2^, reshaped the gut microbiota in colitis-induced mice by increasing the relative abundance of the *Firmicutes* and decreasing the relative abundance of the *Bacteroidota*. Our findings demonstrated that abdominal LIPUS stimulation has the potential to be a novel therapeutic strategy to improve colitis-induced behavioral disorders through microbiota–gut–brain axis signaling.

## Introduction

Inflammatory bowel disease (IBD), which includes Crohn's disease and ulcerative colitis, is associated with anxiety, depression, and altered memory in patients suffering from intestinal inflammation [1]. In recent decades, the incidence and prevalence of IBD have become a challenge to public health worldwide [2]. Many studies have reported that disturbance of the epithelial barrier in the colon results in the invasion of bacterial antigens into the mucosal layer, which triggers the pathogenic mucosal immune system and subsequently leads to immune responses causing damage to the colon [3]. Changes in the gut barrier structure and function might induce inflammation within the brain and further behavioral dysfunctions [4]. Anti-inflammatory or immunosuppressive treatments can be effective therapies, suggesting that inflammation plays a role in the development of behavioral disorders [5].

The intestinal microbiota, mainly consisting of the *Firmicutes*, *Bacteroidetes*, *Actinobacteria*, and *Proteobacteria*, plays an important role in maintaining the integrity and function of barriers, regulation of neurotransmitters, and the immune system [[6], [7], [8]]. An imbalance in the intestinal microbiota can increase lipopolysaccharide (LPS) release and intestinal permeability. This increased permeability can induce increased movement of endotoxins from the gut to the external systemic circulation [9]. LPS also activates immune cells to release inflammatory mediators from the gut and CNS, thereby affecting the blood–brain barrier (BBB) and central nervous system (CNS) [10,11]. Systemic inflammation can disrupt epithelial tight junctions and promote neuroinflammation by triggering the release of various proinflammatory cytokines, such as tumor necrosis factor-α (TNF-α), interleukin-1β (IL-1β), and interleukin-6 (IL-6) [12,13].

Dextran sulfate sodium (DSS)-induced colitis in mice is a robust model of colonic inflammation, with disease symptoms, such as body weight loss, shortened colon, dysbiosis, and behavioral dysfunctions, similar to those observed in patients with ulcerative colitis [14,15]. Numerous studies in animal models of colitis have revealed the presence of inflammatory markers in the hippocampal and cortical areas of the brain [16,17]. These findings are also associated with significantly elevated serum levels of IL-6 and TNF-α in mice with colitis. A growing body of evidence suggests the important role of peripheral infections and intestinal bacterial flora in the physiological functioning of the microbiota–gut–brain axis [18,19]. This axis is a bidirectional communication between the intestine and the brain and has emerged as a potential therapeutic target for the treatment of behavioral disorders. More in-depth investigations into the physiological mechanisms underlying peripheral inflammation and the gut microbiota in neurodegenerative diseases are required to develop novel therapeutic approaches that can weaken the progression of IBD to more advanced steps involving behavioral disorders [20].

Abdominal low-intensity pulsed ultrasound (LIPUS) treatment was recently shown to mitigate the severity of colitis through stimulation of the splenic nerve together with the cholinergic anti-inflammatory pathway [21]. In our previous studies, we demonstrated that abdominal LIPUS could alleviate LPS-induced neuroinflammation via inhibition of colonic inflammation [22,23]. The aim of the present study was, therefore, to explore whether abdominal LIPUS could attenuate colitis and the accompanying behavioral disorders by inhibiting the inflammatory response and reshaping the gut microbiota. Furthermore, we used [^18^F]FEPPA (N-(2-(2-^18^F-fluoroethoxy)benzyl)-N-(4-phenoxypyridin-3-y)acetamide) PET/CT images to simultaneously monitor abdominal and brain inflammation in vivo in DSS-treated mice.

## Materials and Methods

### Animal colitis induction model

Sixty-four male C57BL/6J mice (BioLASCO Taiwan Co., Ltd., Yilan City, Taiwan), aged eight weeks and weighing 22–25 ​g, were used in this study. The mice were housed in a controlled environment with a 12-h light/dark cycle and provided with food ad libitum. The mice were cohoused prior to the experiment to reduce variation associated with cage effects and to promote homogenization of their gut microbiota. Additionally, environmental conditions were controlled by maintaining regular cage cleaning schedules and standardizing bedding and food across all experimental groups. All experimental procedures were conducted in accordance with the guidelines approved by the Animal Care and Use Committee of National Yang Ming Chiao Tung University. Mice were treated with 3 ​% (wt/vol) DSS (molecular weight 36–50 ​kDa; MP Biomedicals Inc., Irvine, CA, USA) dissolved in drinking water for the duration of 7 days. Sham-operated mice received normal water throughout the experimental period.

### Ultrasound system and treatment protocols

LIPUS was generated using a therapeutic ultrasound generator (ME740, Mettler Electronics, Anaheim, CA, USA) coupled with a 1-MHz plane transducer (ME7413, Mettler Electronics) featuring a 4.4-cm^2^ effective radiating area. The ultrasound parameters included 2-ms burst lengths at a 20 ​% duty cycle and a repetition frequency of 100 ​Hz. The spatial average intensity over the plane transducer head was set at either 0.5 ​W/cm^2^ or 1.0 ​W/cm^2^, with measurements obtained using a radiation force balance (RFB, Precision Acoustics, Dorset, UK) in degassed water. The abdominal fur of the mice was shaved prior to LIPUS treatment. Ultrasound transmission gel (Pharmaceutical Innovations, Newark, NJ, USA) was applied to the area between the transducer and the abdomen to maximize ultrasound transmission efficiency. The treatment area encompassed the entire abdomen between the diaphragm and the groin. Each sonication session lasted for 5 ​min, with a 5-min interval between the first and second and between the second and third sonication sessions. Consequently, the total duration of LIPUS stimulation amounted to 15 ​min per day. In this study, we examined the effects of abdominal LIPUS on mice with DSS-induced colitis at the same LIPUS intensities as in our previous studies [22,23]. Mice were randomly allocated into four groups: Sham, DSS, DSS ​+ ​LIPUS 0.5, and DSS ​+ ​LIPUS 1.0. LIPUS treatment was administered consecutively for four days to mice in the DSS ​+ ​LIPUS 0.5 group and DSS ​+ ​LIPUS 1.0 group at ultrasound intensities of 0.5 ​W/cm^2^ and 1.0 ​W/cm^2^, respectively, from day 4 to day 7. After being anesthetized with isoflurane mixed with oxygen, mice in the two LIPUS-treated groups underwent LIPUS stimulation. Mice in the Sham and DSS groups were also anesthetized with isoflurane mixed with oxygen during this period. Body weight changes were monitored daily, and the mice were sacrificed on day 7 after DSS administration to measure colon length. Blood samples were collected via cardiac puncture on day 7. The disease activity index (DAI) score, for assessing clinical symptoms, was calculated as previously described [24]. Mice were observed daily for weight loss, stool consistency, and fecal bleeding to evaluate the severity of colitis. The specific criteria for determining the DAI are outlined in Table S1.

### Real-time polymerase chain reaction

Total RNA was extracted from brain and colonic tissue specimens using the RNeasy Mini Kit (QIAGEN, Germany) in accordance with the manufacturer's protocol. The concentration and purity of the RNA samples were assessed using a spectrophotometer (NanoDrop ND-1000; Thermo Fisher Scientific, Waltham, MA, USA) according to the manufacturer's instructions. Reverse transcription was performed using the ToolsQuant II Fast RT Kit (BioTools, Taiwan) according to the manufacturer's guidelines. RT-PCR analysis was then performed using SYBR Green (KAPA SYBR FAST qPCR Master Mix [2 ​× ​], ABI Prism™, UK) on a StepOnePlus™ real-time PCR system (Applied Biosystems, USA) based on the manufacturer's protocols. Gene-specific expression levels were normalized to GAPDH expression. Each sample was analyzed in triplicate to validate the results. The 2^−ΔΔCt^ method was used for relative quantification of gene expression levels.

### PET/CT imaging and quantification of [^18^F]FEPPA uptake in the abdomen and brain

The labeling efficiency of [^18^F]FEPPA was 75.77 ​% (Fig. S1A), with a non-time-corrected yield of 21.4 ​± ​5.1 ​%. The overall preparation time was 1 ​h. The time-corrected yield was 30.7 ​± ​7.3 ​%. After HPLC purification, 98 ​% radiochemical purity was achieved (Fig. S1B). The retention time (Rt) of purified [^18^F]FEPPA was approximately 10 ​min (Fig. S1C). To observe the levels of inflammation in the abdomen and brain of mice, PET/CT scans were performed for all animals on day 7 after DSS administration. The mice were anesthetized with a mixture of 2 ​% isoflurane and air. Radiotracers were administered by injection into the tail vein at a radioactivity of 18.5 ​MBq (0.5 ​mCi) of [^18^F]FEPPA. After a 30-min uptake of [^18^F]FEPPA, mice were positioned on a nano-PET/CT scanner (Mediso, Budapest, Hungary) for 30 ​min to acquire static distribution images of [^18^F]FEPPA. PET images were reconstructed using the three-dimensional ordered-subset expectation maximization (3D-OSEM) method. The uptake of [^18^F]FEPPA was estimated based on mean pixel values within the three-dimensional volumes of interest (VOIs). For the evaluation of brain inflammation uptake, VOIs of various brain areas were delineated using a built-in mouse brain template of the PMOD image analysis software (version 4.3; PMOD Technologies Ltd., Zurich, Switzerland). In the abdominal region, VOIs were delineated using the auto iso-contour tool of PMOD and PET/CT fusion images, with the diaphragm as the boundary; the whole abdomen was selected to precisely evaluate abdominal inflammation levels (Fig. 3B). The uptake of radioactivity in these brain regions and the abdomen was decay-corrected to the time of injection and quantified as the standard uptake value (SUV), calculated by dividing the radioactivity concentration by the total body concentration of the injected radioactivity, enabling quantitative analysis.

### Enzyme-linked immunosorbent assay

Serum levels of LPS-binding protein (LBP), LPS, and IL-6 were quantified using ELISA kits (LBP [ab269542]: Abcam, Cambridge, UK; LPS [RK04263]: ABclonal, Kirtlington, UK; and IL-6 [M6000B-1]: R&D Systems, MN, USA). The analyses were performed according to the respective manufacturers’ protocol.

### Western blot analysis

Four hours after the final sonication, mice were euthanized for protein expression analysis. Whole protein extracts were prepared from mouse colon tissue by homogenizing it in T-Per extraction reagent supplemented with Halt™ Protease Inhibitor Cocktail (Pierce Biotechnology, Rockford, IL, USA). The lysates were then centrifuged, and the supernatants were collected. Protein concentrations were determined using protein assay reagent (Bio-Rad, CA, USA). Samples containing 30 ​μg of protein were resolved with 12 ​% sodium dodecyl sulfate polyacrylamide gel electrophoresis and transferred to Immun-Blot® polyvinylidene fluoride membranes (Bio-Rad, CA, USA). After blocking for at least 1 ​h in blocking buffer (Hycell, Taipei, Taiwan), the membranes were incubated overnight at 4 ​°C with primary rabbit antibodies against zonula occludens (ZO-1), occludin, and inducible nitric oxide synthase (iNOS). After being washed with PBST buffer, each membrane was incubated with secondary antibodies for 1 ​h at room temperature. The membranes were then washed again with PBST buffer, and signals were developed using Western Lightning ECL Pro reagent (Bio-Rad, CA, USA). Gel images were captured using a biomolecular imager (ImageQuant™ LAS 4000, GE Healthcare Life Sciences, PA, USA) and analyzed using a gel image system (ImageJ) to estimate the integral optical density of the protein bands.

### Histological evaluation of the colon

At the time of sacrifice, which occurred 4 ​h after the final LIPUS treatment, the length of the colon, from the ileocecal junction to the anus, was measured. Distal colon segments were collected, and a longitudinal section (the distal third of the colon from the anal verge) was prepared. These colonic sections were post-fixed overnight in 4 ​% paraformaldehyde and then transferred to phosphate-buffered saline containing 30 ​% sucrose for cryoprotection. Frozen 10-μm sections were coronally cut using a microtome. Hematoxylin and eosin (H&E) staining was performed to observe morphological changes, if any, induced by the DSS and DSS ​+ ​LIPUS treatments. These sections were then analyzed using light microscopy (Nikon E100, Japan) at 100 ​× ​magnification. The colon was scored for various features as previously described [25], including inflammation (0–3), extent (0–3), regeneration (0–4), crypt damage (0–4), and percent involvement (0–4) (Table S2). Each section was individually scored for each feature by calculating the product of the grade for that feature and the percentage involvement.

### Behavioral analysis

All behavioral tests were conducted following a standardized protocol to minimize variability. The same researcher, who was thoroughly trained and blinded to the experimental conditions, administered each test, ensuring consistency and reducing potential bias from variations in test administration. The behavioral experiments were conducted after the final ultrasound treatment session. The open field test (OFT) is commonly used to evaluate depression and anxiety-like behaviors, as previously outlined [26]. Mice were gently placed in a locomotor monitoring box (60 ​cm ​× ​60 ​cm ​× ​30 ​cm) for the duration of 10 ​min. A computerized video-tracking system (EthoVision XT15, Noldus Information Technology, Wageningen, the Netherlands) recorded the total distance of spontaneous movement and the average speed of the mice during this time. For the novel object recognition task (NORT), mice were first habituated in individual cages for 1 ​h. Following habituation, mice underwent both a training phase and a testing phase to assess recognition memory. Exploratory behavior during these phases was recorded using a video camera for 10 ​min each. Video recordings were analyzed to determine the number of times mice approached or sniffed each object, and the results were expressed as an exploration ratio. Mice were considered to have investigated an object when their nose came within 1–2 ​cm of the object. Mice that did not pass the training phase or did not approach the objects during the testing phase were excluded from the analysis. A mouse with unimpaired memory was expected to preferentially explore the new object, resulting in an exploration ratio greater than 50 ​% [27]. Spatial recognition memory was assessed using the Y-maze paradigm. Mice were individually habituated to a Y-shaped maze (30 ​cm height ​× ​16 ​cm length ​× ​8 ​cm width) for 15 ​min each without any objects in the arms. Subsequently, with the novel arm sealed off, mice were placed into the start arm to explore for 15 ​min. After a 30-min interval, mice were reintroduced into the same arm for 5 ​min of uninterrupted exploration with the divider removed. Arm entry times were recorded and analyzed using a video tracking system (EthoVision XT15, Noldus Information Technology, Wageningen, the Netherlands) when all four paws of the mouse were inside the arm. The percentage of time spent in the novel arm was calculated as the novel arm exploration time divided by the total exploration time [28].

### Analysis of the gut microbiota

Total genomic DNA was extracted from frozen fecal samples using the CatchGene™ Stool DNA kit. The DNA concentration was quantified using a Qubit 4.0 fluorometer (Thermo Scientific) and adjusted to 1 ​ng/μl for subsequent processing. Amplification of the full-length 16S gene (V1–V9 regions) was performed using barcoded 16S gene-specific primers. These primers, designed for multiplexed SMRTbell library preparation and sequencing on the PacBio platform, included a 5′ buffer sequence (GCATC) with a 5′ phosphate modification, a 16-base barcode, and degenerate 16S gene-specific forward or reverse primer sequences. The amplified sequences were clustered into operational taxonomic units (OTUs) using a 97 ​% similarity threshold. Beta diversity analysis, employing principal coordinate analysis (PCoA), was performed to assess differences in species complexity between samples. Differential abundance analysis was performed using a zero-inflated Gaussian log-normal model implemented in the “fitFeatureModel” function of the Bioconductor metagenomeSeq package to detect significant differences in taxa between the groups. In addition, Welch's *t*-test was applied using STAMP software. Statistically significant biomarkers were identified using the linear discriminant analysis (LDA) effect size (LEfSe) algorithm. LEfSe uses non-parametric tests such as the Kruskal–Wallis test and Wilcoxon rank-sum test to identify bacterial taxa with significantly different relative abundances between the control and the group of interest. The algorithm then applies LDA to evaluate the effect size of each differentially abundant taxon. Taxa with LDA scores (log 10) exceeding 4 were considered statistically significant in this study.

### Statistical analysis

All data are presented as means ​± ​standard error of the mean (SEM). All data were assessed for normal distribution using the Kolmogorov-Smirnov test. Statistical analyses were performed using one-way ANOVA for all data followed by a post-hoc Tukey's test to identify significant differences between groups (GraphPad Prism 6, GraphPad Inc., La Jolla, CA, USA). A p-value of less than 0.05 was considered statistically significant.

## Results

### Abdominal LIPUS stimulation alleviated DSS-induced colitis in mice

**Fig. 1:**
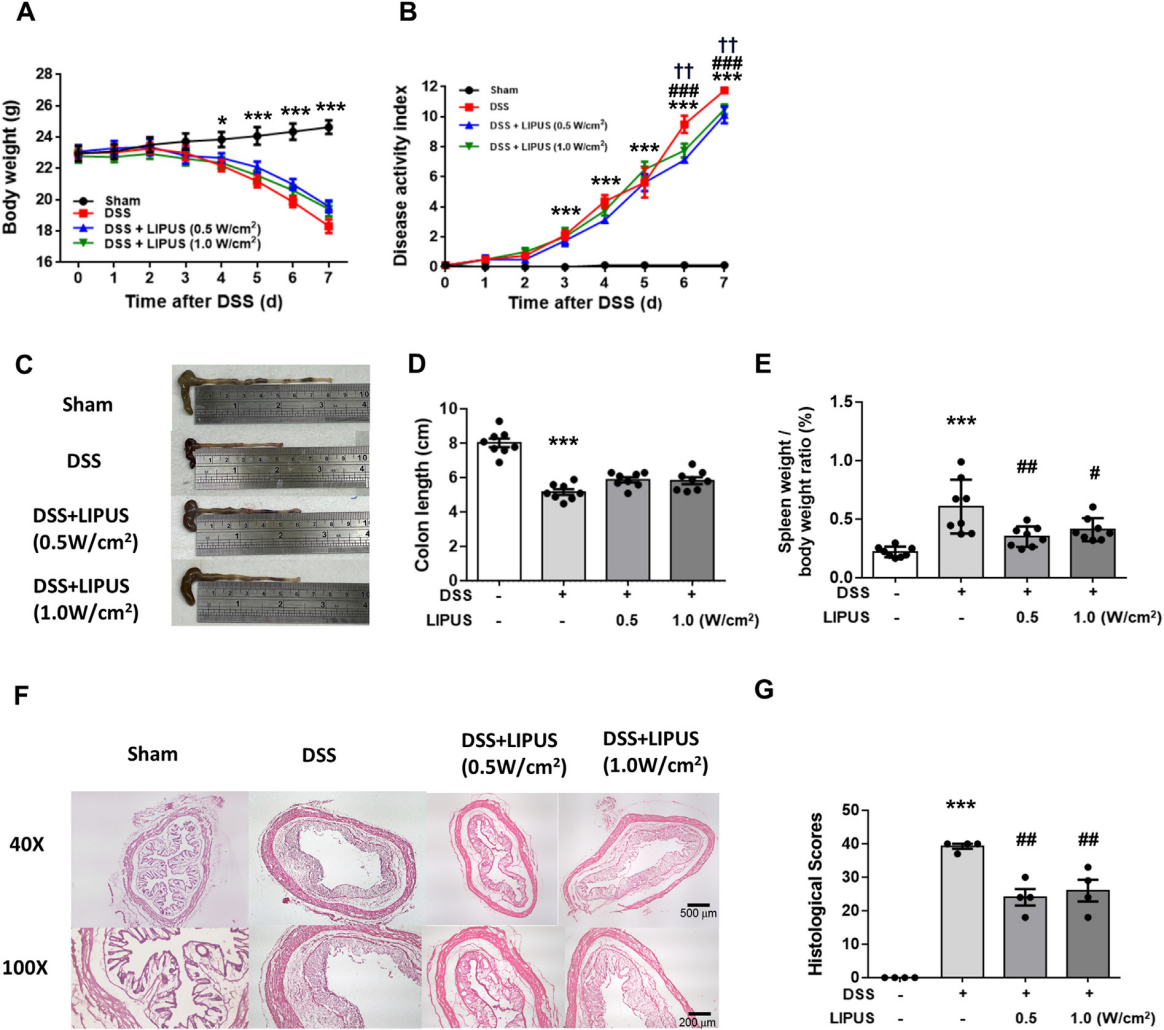
LIPUS mitigated colon damage in DSS-induced acute colitis in mice. (A) Changes in body weight in different groups. (B) The disease activity index (DAI) score for each group. (C) Representative images of the colon. (D) Measurement of colon length. LIPUS reduced the increase in the spleen weight/body weight ratio in DSS-induced acute colitis. (F) Representative hematoxylin and eosin–stained images of the colon. (G) Histological score of each group. In (A) and (B), ∗, ^#^, and ^†^ denote a significant difference between the DSS group and the Sham group, DSS ​+ ​LIPUS 0.5 group, and DSS ​+ ​LIPUS 1.0 group, respectively. In (C), (D), and (E), ∗ and ^#^ denote significant differences from the Sham group and the DSS group, respectively (∗^,#^, *p* ​< ​0.05; ^##,††^, *p* ​< ​0.01; ∗∗∗^,###^, *p* ​< ​0.001; n ​= ​6–8).

The mice treated only with DSS showed a more significant loss of body weight (Fig. 1A) and the highest DAI score (Fig. 1B) compared to the other groups. LIPUS treatment resulted in a significant reduction in the DAI score at ultrasound intensities of 0.5 ​W/cm^2^ (*p ​<* ​0.001) and 1.0 ​W/cm^2^ (*p ​<* ​0.01) by day 7 compared to DSS treatment alone (Fig. 1B). The colon length of mice in the DSS-only group was significantly shorter than that in the Sham group (*p ​<* ​0.001; Fig. 1C and D). The colon length of mice in both LIPUS treatment groups was not significantly different from that of mice in the DSS-only group. The ratio of spleen weight/body weight was significantly greater in the DSS-only group compared to the Sham group (*p ​<* ​0.001; Fig. 1E). However, the spleen weight/body weight ratios were significantly reduced in mice treated the LIPUS at intensities of 0.5 ​W/cm^2^ (*p ​<* ​0.01) and 1.0 ​W/cm^2^ (*p ​<* ​0.05) (Fig. 1E). Pathological alterations in colonic tissue were observed after H&E staining. Histological analysis indicated a significant reduction in DSS-induced colonic damage, characterized by lower crypt destruction and partial preservation of the epithelial barrier in the DSS ​+ ​LIPUS 0.5 group and DSS ​+ ​LIPUS 1.0 group compared to the DSS-only group (*p* ​< ​0.01; Fig. 1F and G).

### Abdominal LIPUS attenuated DSS-induced inflammatory cytokines in the mouse colon and brain

**Fig. 2:**
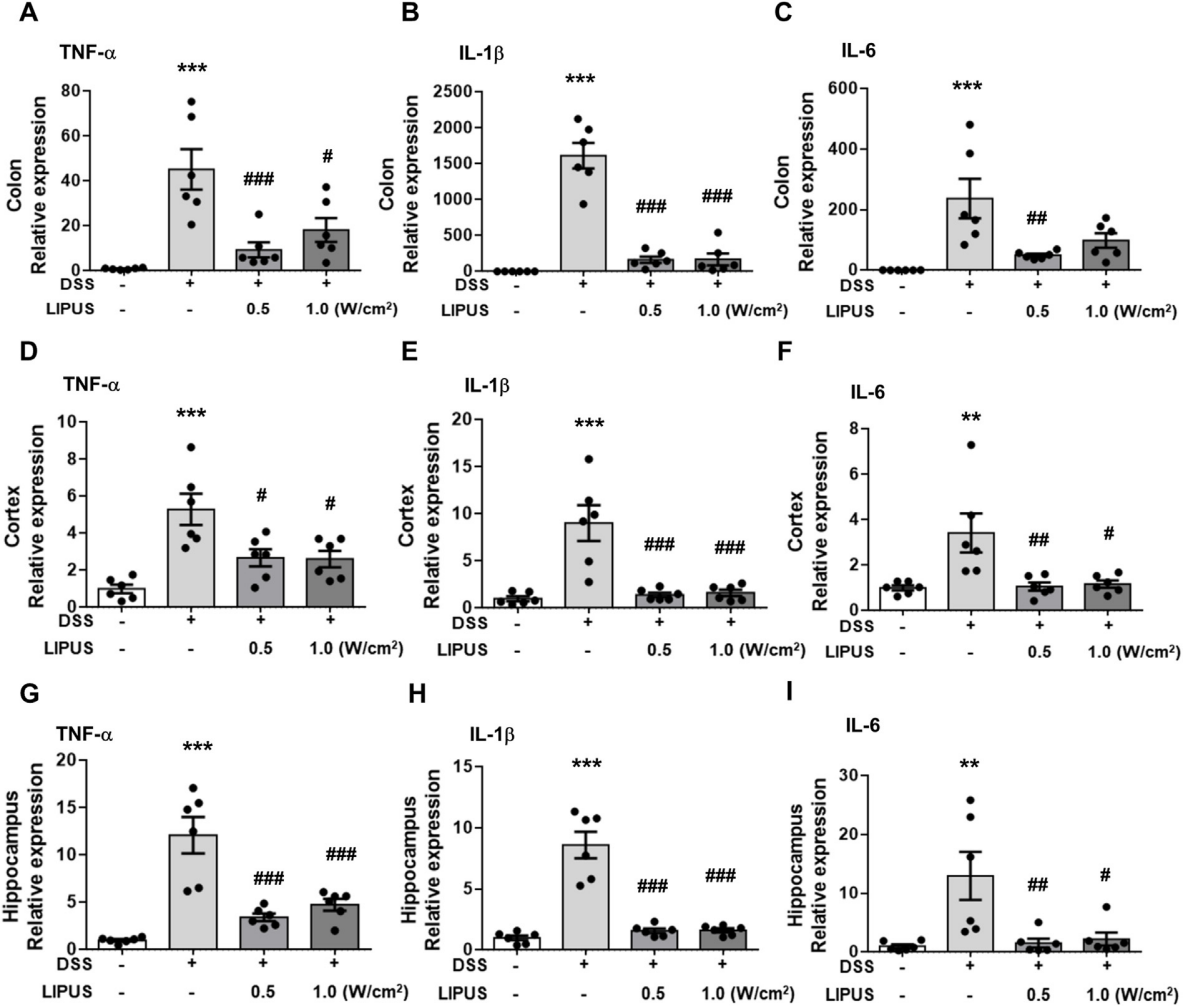
LIPUS inhibited inflammatory cytokine expression in DSS-induced acute colitis in mice. Quantification of the mRNA levels of (A) TNF-α, (B) IL-1β, and (C) IL-6 in colon tissue using qRT-PCR. Quantification of the mRNA levels of (D) TNF-α, (E) IL-1β, and (F) IL-6 in the cortex using qRT-PCR. Measurement of the mRNA levels of (G) TNF-α, (H) IL-1β, and (I) IL-6 in the hippocampus using qRT-PCR.∗, ^#^, and ^†^ denote significant difference from the Sham group, DSS group, and DSS ​+ ​LIPUS 0.5 group, respectively (^#^, *p* ​< ​0.05; ^##^, *p* ​< ​0.01; ∗∗∗^,###^, *p* ​< ​0.001; n ​= ​6).

DSS administration led to a significant increase in the expression of TNF-α, IL-1β, and IL-6 in colon tissue (all *p ​<* ​0.001; Fig. 2A–C). The expression of TNF-α, IL-1β, and IL-6 was significantly reduced in mice treated with LIPUS compared to mice treated only with DSS (all *p ​<* ​0.05; Fig. 2A–C). In parallel, DSS administration led to a significant rise in TNF-α, IL-1β, and IL-6 expression in the cortex and hippocampal region of the mice (all *p ​<* ​0.01; Fig. 2D–I). Subsequent application of LIPUS at both intensities resulted in a significant decrease in TNF-α, IL-1β, and IL-6 expression levels (all *p* ​< ​0.05; Fig. 2D–I). These findings suggest that abdominal LIPUS stimulation exerted anti-inflammatory effects on DSS-induced intestinal and brain inflammation.

**Fig. 3:**
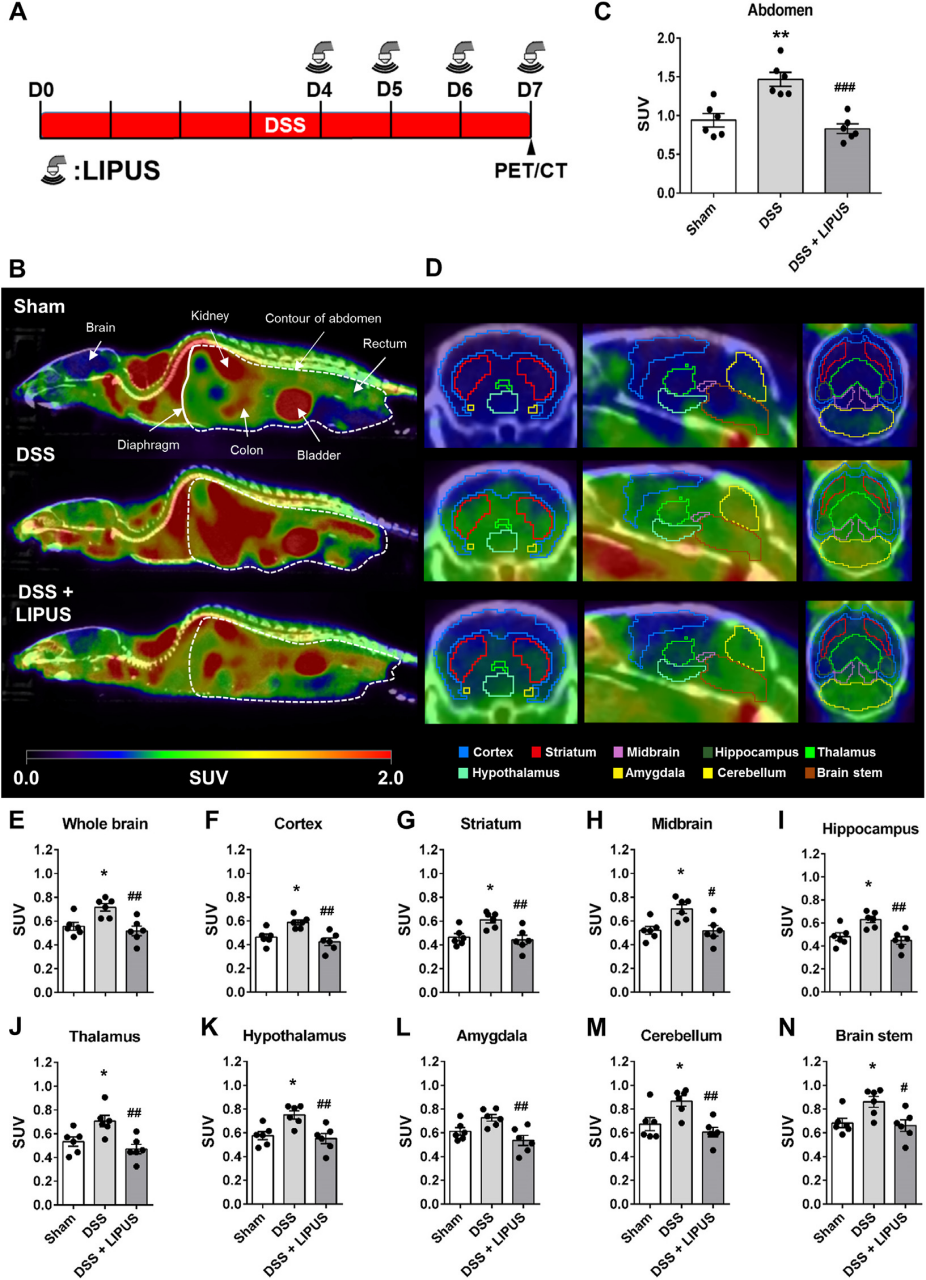
PET/CT imaging evaluated the effects of abdominal LIPUS stimulation on the level of inflammation in the abdomen and brain of mice. (A) Mice were given DSS for 7 days to induce an IBD disease model. Abdominal LIPUS stimulation was performed from day 4 to day 7. A PET/CT scan was performed on day 7 to evaluate the inflammation levels after LIPUS treatment (DSS: dextran sulfate sodium; PET: positron emission tomography; CT: computed tomography). (B) Representative PET/CT images of [^18^F]FEPPA were obtained for the Sham, DSS, and DSS ​+ ​LIPUS groups on day 7. PET/CT images of the levels of inflammation in the abdomen and brain were obtained simultaneously. The white-dashed area shows the contour of the abdomen for quantitative analysis. (C) Results of quantitative analysis of the averaged standard uptake value (SUV) of [^18^F]FEPPA in the abdomen showed that abdominal LIPUS stimulation improved DSS-induced abdominal inflammation in the DSS ​+ ​LIPUS group compared to the DSS group. The SUV of each group is color-coded within the specified range. These images show the levels of inflammation after LIPUS treatment. (D) PET/CT images of the brain were obtained 30 ​min after intravenous injection of [^18^F]FEPPA. The different colored lines indicate the contours of the respective brain sections used for quantitative analysis. Quantitative analysis of [^18^F]FEPPA uptake in (E) the whole brain, (F) cortex, (G) striatum, (H) midbrain, (I) hippocampus, (J) thalamus, (K) hypothalamus, (L) amygdala, (M) cerebellum, and (N) brain stem. [^18^F]FEPPA uptake was lower in all brain regions after abdominal LIPUS stimulation compared to the uptake in the DSS group. ∗ and ^#^ denote significant differences from the Sham group and DSS group, respectively (∗,^#^, *p* ​< ​0.05; ∗∗^,##^, *p* ​< ​0.01; ^###^, *p* ​< ​0.001; n ​= ​6).

### In vivo uptake of [^18^F]FEPPA in the abdomen and brain regions

The whole-body PET/CT images in Fig. 3 show the [^18^F]FEPPA signals in the Sham, DSS, and DSS ​+ ​LIPUS groups. Abdominal PET/CT imaging (Fig. 3B) revealed that after DSS administration, the abdominal uptake of [^18^F]FEPPA was higher in the DSS group compared to the Sham group. After abdominal LIPUS stimulation, the [^18^F]FEPPA uptake in the DSS ​+ ​LIPUS group was lower than that in the DSS group. By averaging the SUV of each voxel in the abdominal images to quantify the inflammation level, the [^18^F]FEPPA SUV of the DSS group was significantly higher than that of the Sham group (Fig. 3C; 1.47 ​± ​0.09 ​g/ml vs. 0.41 ​± ​0.09 ​g/ml; *p* ​= ​0.0011). On day 7 after DSS administration, the SUV of the DSS ​+ ​LIPUS group was lower by 43.5 ​% relative to that of the DSS group (0.83 ​± ​0.06 ​g/ml vs. 1.47 ​± ​0.09 ​g/ml; *p* ​= ​0.0002). These findings from PET/CT imaging and quantification results indicate that abdominal LIPUS stimulation alleviated DSS-induced abdominal inflammation.

The PET/CT imaging in Fig. 3D shows that the [^18^F]FEPPA uptake in the brain was higher in mice administered DSS for 7 days compared to the Sham mice. This result indicates that DSS not only induced inflammation in the abdomen, but also caused neuroinflammation in the brain of mice. However, PET/CT imaging revealed a lower level of neuroinflammation in the DSS ​+ ​LIPUS group, which received LIPUS stimulation to the abdomen, compared to the DSS group. Quantitative analysis of the mean SUV of the whole brain also showed that abdominal LIPUS stimulation could significantly alleviate DSS-induced brain inflammation (Fig. 3E; 0.518 ​± ​0.04 ​g/ml vs. 0.715 ​± ​0.03 ​g/ml; *p* ​= ​0.0034). Using the brain templates of PMOD software, different brain regions (Fig. 3F–N), including the cortex, striatum, midbrain, hippocampus, thalamus, hypothalamus, amygdala, cerebellum, and brain stem, could be delineated, and their SUVs could be calculated for quantitative analysis. In the Sham group under normal conditions, we found a relatively higher uptake of [^18^F]FEPPA in the amygdala, cerebellum, and brain stem. Compared to the Sham group, there was a significant increase in [^18^F]FEPPA uptake in all brain regions of the DSS group after the administration of DSS for 7 days (cortex, *p* ​= ​0.0147; striatum, *p* ​= ​0.0162; midbrain, *p* ​= ​0.0143; hippocampus, *p* ​= ​0.0138; thalamus, *p* ​= ​0.0335; hypothalamus, *p* ​= ​0.0131; cerebellum, *p* ​= ​0.0304; and brain stem, *p* ​= ​0.0395), except for the amygdala (Fig. 3L; *p* ​= ​0.1046). After 4 days of abdominal LIPUS stimulation, the DSS ​+ ​LIPUS group showed a significant decrease in [^18^F]FEPPA SUVs in all brain regions compared to the DSS group (cortex, *p* ​= ​0.0018; striatum, *p* ​= ​0.006; midbrain, *p* ​= ​0.0121; hippocampus, *p* ​= ​0.0031; thalamus, *p* ​= ​0.0041; hypothalamus, *p* ​= ​0.0051; amygdala *p* ​= ​0.0046; cerebellum, *p* ​= ​0.0037; and brain stem, *p* ​= ​0.0002). Following LIPUS stimulation of the abdomen, inflammation in various brain regions tended to decrease. In vivo PET/CT imaging confirmed that abdominal LIPUS stimulation alleviated DSS-induced inflammation in both the abdomen and the brain.

### Abdominal LIPUS attenuated intestinal barrier leakage in mice with colitis

**Fig. 4:**
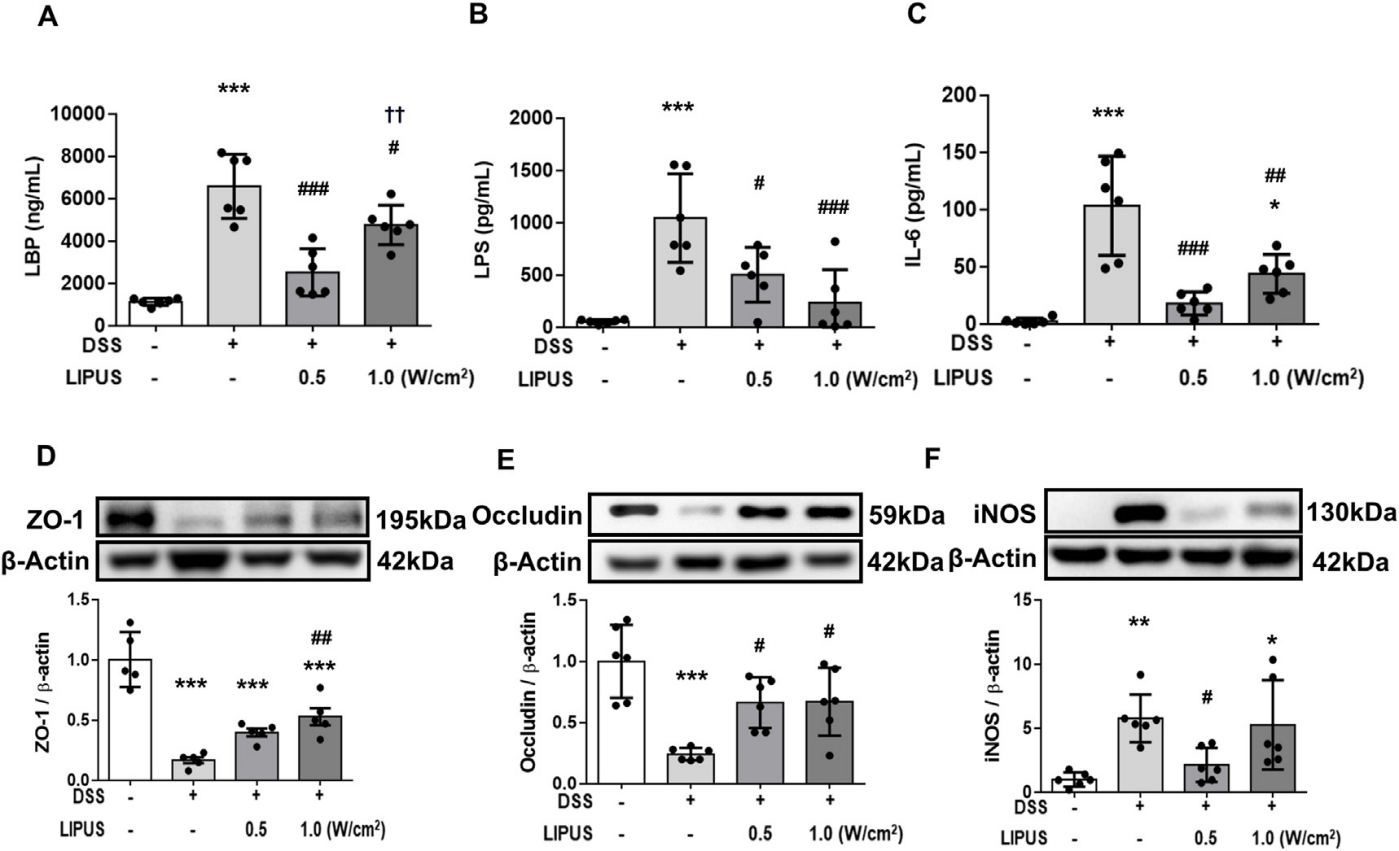
LIPUS inhibited the neuroinflammatory response and alleviated tight junction protein degradation in the colon of mice with DSS-induced acute colitis. Serum levels of (A) lipopolysaccharide-binding protein (LBP), (B) lipopolysaccharide (LPS), and (C) interleukin-6 (IL-6) were measured. The relative levels of (D) zonula occludens (ZO-1), (E) occludin, and (F) inducible nitric oxide synthase (iNOS) in colon tissue were assessed. ∗, ^#^, and ^†^ denote significant differences from the Sham group, DSS group, and DSS ​+ ​LIPUS 0.5 group, respectively (∗,^#^, *p* ​< ​0.05; ^##,††^, *p* ​< ​0.01; ∗∗∗^,###^, *p* ​< ​0.001; n ​= ​6).

The levels of LBP, LPS, and IL-6 in serum were analyzed (Fig. 4A–C). The assessment of LBP and LPS levels in serum serves as an indicator of intestinal barrier integrity. Remarkably, the levels of LBP and LPS were significantly elevated in the DSS-treated mice (both *p* ​< ​0.001; Fig. 4A and B), reflecting the increased burden of bacteria-derived antigens. However, this elevation was significantly attenuated following LIPUS treatment at both 0.5 ​W/cm^2^ and 1.0 ​W/cm^2^ (both *p ​<* ​0.05; Fig. 4A and B), indicating a protective effect of LIPUS on intestinal barrier function. Furthermore, serum IL-6 upregulation, indicative of systemic inflammation triggered by intestinal inflammation, was observed in the DSS group (*p ​<* ​0.001; Fig. 4C). Notably, the serum IL-6 level showed a significant decrease after LIPUS treatment at both intensities compared to the level in the DSS group (both *p ​<* ​0.01; Fig. 4C). We further investigated the impact of LIPUS on the expression of two key proteins, ZO-1 and occludin, which are critical components of the tight junctions in the gut barrier. DSS treatment resulted in a significant reduction in the levels of both ZO-1 and occludin (both *p* ​< ​0.001; Fig. 4D and E). However, LIPUS administration at an intensity of 1.0 ​W/cm^2^ significantly restored ZO-1 and occludin expression compared to the expression in the DSS group (*p* ​< ​0.05; Fig. 4D and E). In addition, increased iNOS levels in the colon were indicative of intestinal inflammation in the DSS group (*p ​<* ​0.001; Fig. 4F). Notably, compared to the DSS-only treatment, LIPUS treatment at both intensities led to a significant reduction in iNOS expression (both *p* ​< ​0.05; Fig. 4F). These findings suggest that LIPUS treatment played a role in restoring the integrity of the intestinal barrier and alleviating the systemic inflammation triggered by DSS administration.

### Abdominal LIPUS alleviated DSS-induced behavioral dysfunction

**Fig. 5:**
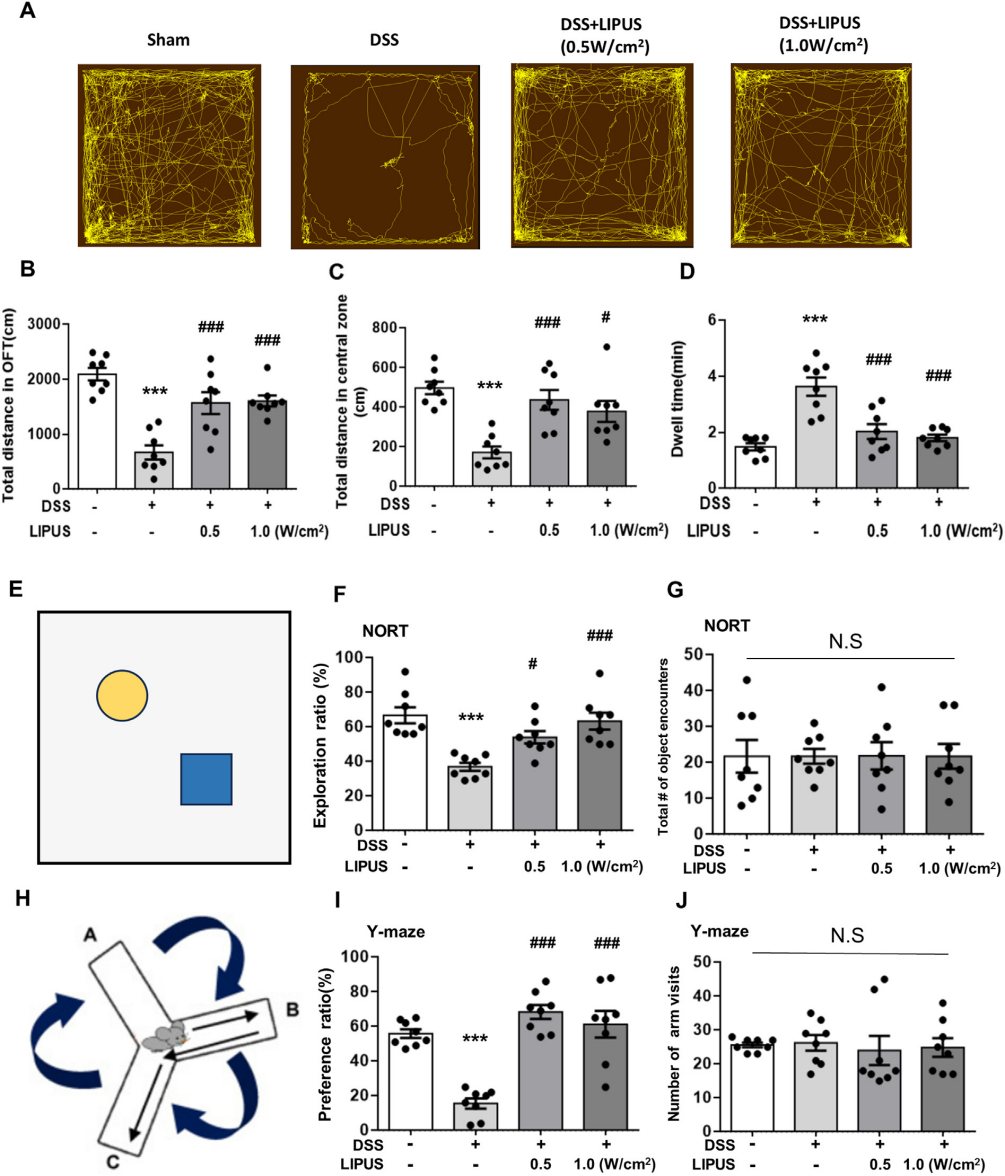
LIPUS alleviated DSS-induced behavioral disorders in mice. (A) Representative movement paths in the open field test (OFT). (B) Total distance traveled in the entire OFT area. (C) Distance traveled in the central OFT area. (D) Dwell time results. (E) The novel object recognition task (NORT). (F) Exploration ratios of the four groups. DSS-treated mice exhibited significant object memory impairment compared to Sham mice, while LIPUS treatments ameliorated these deficits. (G) The number of object encounters in the four groups. (H) The Y-maze test. (I) Preference ratios of the four groups. DSS-treated mice had a reduced preference ratio compared to Sham mice; the preference ratio was significantly improved by LIPUS treatment. (J) The total number of arm visits in the four groups. ∗, ^#^, and ^†^ denote significant differences from the Sham group, DSS group, and DSS ​+ ​LIPUS 0.5 group, respectively (^#^, *p* ​< ​0.05; ∗∗∗^, ###^, *p* ​< ​0.001; n ​= ​8–10).

The OFT is a commonly employed method to evaluate depression and anxiety-like behaviors in mice (Fig. 5A). The total distances moved by mice in the DSS group were significantly reduced in both the entire OFT area and the central zone compared to the distances moved by the Sham group (both *p* ​< ​0.001; Fig. 5B and C). Interestingly, mice in both LIPUS groups demonstrated a substantial increase in the total distance moved across the entire area compared to the DSS group (both *p ​<* ​0.001; Fig. 5B). Moreover, LIPUS treatment at both intensities led to a significant increase in the total distance moved across the central area compared to the DSS-only treatment (both *p ​<* ​0.05; Fig. 5C). Analysis of dwell time revealed depressive and anxiety-like behaviors of DSS-treated mice (Fig. 5D), characterized by an extended dwell time (p ​< ​0.001). Notably, LIPUS treatment at both intensities significantly mitigated this behavioral dysfunction compared to the DSS-only treatment (both *p* ​< ​0.001; Fig. 5D).

The NORT was employed to evaluate recognition memory (Fig. 5E). DSS-treated mice exhibited impairments in recognition memory, evidenced by a significantly lower exploration ratio, compared to the Sham group (*p ​<* ​0.001; Fig. 5F). Treatment with LIPUS at both intensities significantly ameliorated recognition memory deficits in mice with DSS-induced colitis compared to treatment with DSS only (both *p* ​< ​0.05; Fig. 5F). The four groups did not show significant differences in the total number of object encounters (Fig. 5G). In addition, spatial recognition memory was measured using the Y-maze test (Fig. 5H). The DSS-treated mice displayed impaired spatial recognition memory, as indicated by a significantly lower preference ratio (*p ​<* ​0.001; Fig. 5I). LIPUS treatment at both intensities significantly improved spatial memory deficits compared to the DSS-only treatment (both *p* ​< ​0.001; Fig. 5I). The total number of arm visits did not differ significantly between the four groups, suggesting that DSS-induced colitis and DSS plus LIPUS treatment did not affect locomotor activity (Fig. 5J).

### Effects of abdominal LIPUS on the taxonomic composition of the intestinal microbiota in mice with DSS-induced colitis

**Fig. 6:**
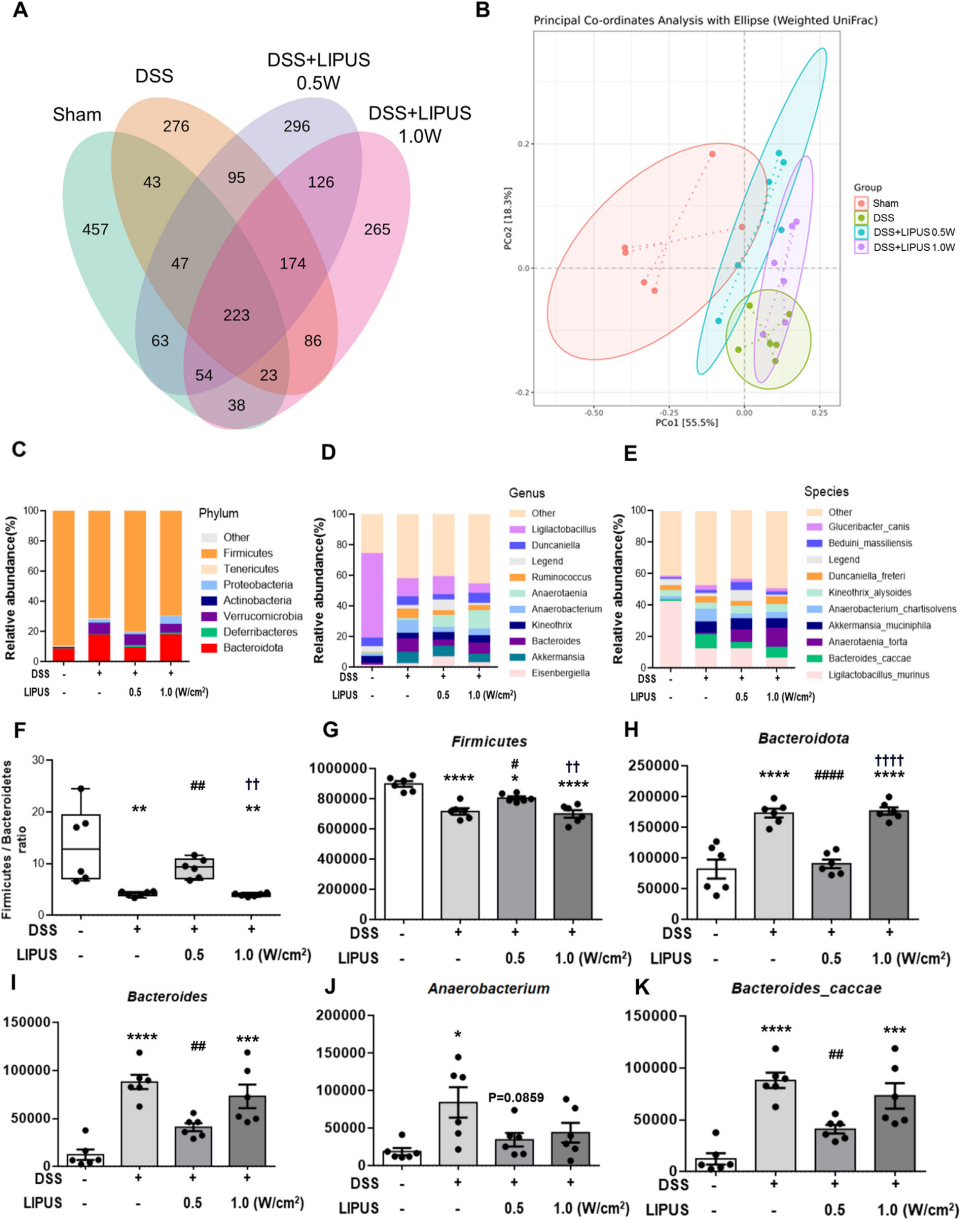
Effects of LIPUS treatment on the gut microbiota structure in mice with DSS-induced colitis. (A) A Venn diagram was used to assess the shared and group-specific operational taxonomic units of the four groups. (B) Principal coordinate analysis (PCoA) based on the Bray–Curtis distance of the four groups. Each symbol represents an individual mouse. Relative abundance bar plots show bacterial composition at the (C) phylum, (D) genus, and (E) species level of each group. (F) The ratio of *Firmicutes* to *Bacteroidota* of the four groups at the phylum level. LIPUS significantly increased the F/B ratio at an intensity of 0.5 ​W/cm^2^. (G) The phylum *Firmicutes*. (H) The phylum *Bacteroidota*. (I) The genus *Bacteroides.* (J) The genus *Anaerobacterium*. (K) The species *Bacteroides caccae*. ∗, ^#^, and ^†^ denote significant differences from the Sham group, DSS group, and DSS ​+ ​LIPUS 0.5 group, respectively (∗,^#^, *p* ​< ​0.05; ∗∗^,##,††^, *p* ​< ​0.01; ∗∗∗, *p* ​< ​0.001; ∗∗∗∗^,####,††††^, *p* ​< ​0.0001; n ​= ​6).

The relative proportions of the microbiotic taxa were evaluated at phylum, genus, and species levels to investigate changes in composition (Fig. 6). The number of distinct OTUs values in each group exceeded 265, with 223 OTUs values shared between all groups, as shown in Fig. 6A. PCoA was employed to visualize β-diversity and discern disparities in microbial community structure between multiple sample groups (Fig. 6B). The distance between groups indicated the extent of dissimilarity. We found noticeable separation between the Sham and DSS groups. However, the microbial composition of groups treated with LIPUS at the intensities of 0.5 ​W/cm^2^ and 1.0 ​W/cm^2^ was more similar to that of the Sham group and the DSS group, respectively. Fig. 6C shows microbial composition at the phylum level. The predominant bacterial phyla observed in the Sham group were the *Firmicutes* (89.95 ​%) and *Bacteroidota* (8.24 ​%). In contrast, DSS treatment had a significant effect on the relative abundances of these taxa, causing a reduction in the *Firmicutes* to 71.74 ​% and an increase in the abundance of the *Bacteroidota* to 17.34 ​% and the *Verrucomicrobia* to 7.33 ​% (compared to 0.00 ​% in the Sham group). Consistent with previous reports, our DSS-treated mice indicated that the effects on gut microbes mainly manifested as a decrease in the *Firmicutes* and an increase in the *Proteobacteria* compared to the effects in Sham mice [4,29]. The relative abundances of the *Firmicutes* (80.65 ​%) and *Bacteroidota* (9.09 ​%) in the DSS ​+ ​LIPUS 0.5 group were similar to those in the Sham group. In contrast, the relative abundances of the *Firmicutes* (70.11 ​%) and *Bacteroidota* (17.68 ​%) in the DSS ​+ ​LIPUS 1.0 group were similar to those of the DSS group.

At the genus level, DSS-induced colitis resulted in a significant reduction in the abundance of *Ligilactobacillus* (Fig. 6D), accompanied by elevated levels of *Bacteroides*, *Akkermansia*, *Anaerobacterium*, and *Ruminococcus*. LIPUS treatment led to a rise in the relative abundance of *Anaerotaenia* and a reduction in the abundances of *Anaerobacterium* and *Ruminococcus*. Furthermore, there was no marked difference in taxonomic composition and proportion at the genus and species level between the four groups (Fig. 6D and E). A decrease in the *Firmicutes* to *Bacteroidota* (F/B) ratio, resulting in gut microbiota dysbiosis, is associated with IBD [30]. Compared to the mice in the Sham group, those subjected to DSS treatment exhibited a significant decrease in the F/B ratio (*p ​<* ​0.01; Fig. 6F). The F/B ratio significantly increased following treatment with LIPUS at 0.5 ​W/cm^2^ compared to treatment with DSS only (*p ​<* ​0.01; Fig. 6F). However, no significant difference was found in the F/B ratio between the DSS group and the DSS ​+ ​LIPUS 1.0 group. The relative abundance of the *Firmicutes* was significantly reduced in DSS-treated mice (*p ​<* ​0.001; Fig. 6G). The relative abundance of the *Firmicutes* was significantly higher in mice treated with LIPUS at an intensity of 0.5 ​W/cm^2^ (*p* ​< ​0.05) compared to mice treated only with DSS, whereas no significant difference was observed at an intensity of 1.0 ​W/cm^2^. In contrast, the relative abundances of *Bacteroidota* increased significantly in DSS-treated mice at the phylum, genus, and species level (all *p ​<* ​0.001; Fig. 6H, I, K). The relative abundances of the *Bacteroidota* were significantly reduced in mice treated with LIPUS at an intensity of 0.5 ​W/cm^2^ (all *p ​<* ​0.01) compared to the mice treated with DSS only, whereas no significant difference was observed at an intensity of 1.0 ​W/cm^2^. In addition, the relative abundance of the genus *Anaerobacterium* was significantly higher in DSS-treated mice compared to the Sham mice (*p ​<* ​0.05; Fig. 6J). There was a non-significant decreasing trend in the relative abundance of the genus *Anaerobacterium* in both LIPUS treatment groups compared to the DSS group.

## Discussion

Recent investigations have shown that individuals with IBD are at increased risk of developing psychological and cognitive deficits, including depression and anxiety, compared to healthy controls. However, the underlying mechanisms linking intestinal inflammation to behavioral disorders remain largely unknown [31,32]. In the present study, abdominal LIPUS was used to treat DSS-induced colitis in mice. Our findings showed that abdominal LIPUS could not only reduce proinflammatory cytokines in the colon, but also mitigate inflammatory responses in the brain. In addition, LIPUS could reduce gut barrier damage and serum LPS and LBP levels. Abdominal LIPUS also alleviated behavioral disorders after exposure to DSS. Furthermore, abdominal LIPUS clearly altered the gut microbiota composition in DSS-induced colitis. These findings indicate that abdominal LIPUS can attenuate DSS-induced colitis, neuroinflammation, and the associated behavioral disorders, potentially through mechanisms involving gut–brain communication.

Ultrasound treatment, depending on its intensity and duration, can have varying effects on microbial cells [33]. This noninvasive physical stimulus has potential in various therapeutic applications [34]. LIPUS, a form of acoustic energy administered at low intensity through pulsed waves, can stimulate bacterial metabolism [35]. Bacterial adhesion may be strongly influenced by ultrasound treatment [36]. It is reasonable to assume that LIPUS could promote the release of cellular components and increase the rate of exchange with the environment, thereby enhancing microbial growth [37,38]. On the other hand, the administration of LIPUS to the mouse abdomen alleviates colitis by stimulating the splenic nerve [21]. Abdominal LIPUS mitigates the LPS-induced elevation of TNF-α, IL-1β, and IL-6 in both the colon and the brain [22]. Our findings align with those of previous studies supporting the hypothesis that abdominal LIPUS can mitigate neuroinflammation by reducing gut inflammation, without the need for direct brain stimulation. Therefore, abdominal LIPUS stimulation can circumvent the obstacle of ultrasound penetration through the skull and simultaneously treat brain-related diseases triggered by intestinal inflammation.

This study is the first to use [^18^F]FEPPA PET/CT imaging to simultaneously investigate DSS-induced abdominal and brain inflammation. PET offers a significant advantage over other functional imaging techniques due to its ability to quantify absolute regional radiotracer concentrations [39,40]. Activated leukocytes show significant accumulation of [^18^F]FDG, rendering it detectable through PET imaging [41,42]. The translocator protein (TSPO), previously known as the peripheral benzodiazepine receptor (PBR), is an 18 ​kDa protein primarily localized on the outer membrane of mitochondria [43]. Increased TSPO expression is widely recognized as a hallmark of inflammation, largely due to its association with microglial activation, making it a critical target for imaging in positron emission tomography (PET) studies of both the central and peripheral nervous systems [[44], [45], [46]]. The second-generation PET radiotracer [^18^F]FEPPA, which targets TSPO, offers significant advantages over the first-generation tracer [^11^C]PK11195. Specifically, [^18^F]FEPPA demonstrates higher specificity and affinity, resulting in a superior signal-to-noise ratio and improved image resolution, particularly for detecting neuroinflammation. Previous research has employed [^18^F]FEPPA to assess brain inflammation following intraperitoneal LPS administration [47] and in patients with Parkinson's disease [48]. Thus, we consider [^18^F]FEPPA an ideal radiotracer for simultaneously monitoring inflammation in both the brain and abdomen in our study.

The DSS-induced acute colitis model is commonly used to investigate early inflammatory responses, epithelial barrier dysfunction, and activation of the innate immune system [49]. While chronic colitis models more accurately represent the prolonged progression of IBD, the acute model provides important insights into short-term changes in barrier function and the initial immune responses that may trigger neuroinflammatory cascades [50]. Since acute inflammatory flares can potentially initiate neurodegeneration, particularly in the context of IBD, this model serves as a valuable system for studying the early mechanisms connecting gut inflammation to neurodegenerative processes.

To investigate whether DSS-induced abdominal inflammation triggers neuroinflammation in the brain via the gut–brain axis and to assess whether abdominal LIPUS stimulation can alleviate brain inflammation, we employed high-sensitivity in vivo PET/CT imaging. The application of PET/CT imaging with [^18^F]FEPPA has its limitations. In previous studies, imaging segmentation of the brain, lungs, heart, kidneys, bladder, and skeleton was facilitated by image analysis algorithms to enable automated quantification. However, soft tissues such as the colon, which undergo changes over time, pose a challenge to PET/CT image segmentation and quantification due to their relatively low contrast [39]. Without the use of contrast agents, the accurate identification of the location of the colon in CT images is a challenge. Furthermore, we found that DSS not only induced inflammation in the colon, but also affected surrounding tissues. Consequently, we defined the contour as the entire abdominal area below the diaphragm to represent DSS-induced abdominal inflammation. Future studies should consider employing PET/MR imaging for precise identification of soft tissue locations.

Our observations confirm the notion that colitis disrupts the composition of the gut microbiota and elevates the levels of proinflammatory mediators [51,52]. The ratios of major bacterial phyla are widely recognized to exert an important influence on the composition of the gut microbiota [53]. To the best of our knowledge, this study is the first to report the impact of abdominal LIPUS stimulation on the gut microbiota in DSS-induced colitis. Overall, we found that the F/B ratio was significantly elevated in DSS-treated mice after abdominal LIPUS stimulation at an intensity of 0.5 ​W/cm^2^ (*p* ​< ​0.01; Fig. 6F), whereas we did not observe a significant change at an intensity of 1.0 ​W/cm^2^. Our results are consistent with previous findings indicating that the *Firmicutes* possess anti-inflammatory effects and may mitigate the advancement of IBD (Fig. 6G). In addition, the *Bacteroidota* display pro-inflammatory characteristics attributed to endotoxins, which affect cytokine production and contribute to the pathogenesis of IBD (Fig. 6H, I, K) [53]. Furthermore, the gut microbiota modulates host immunity by producing microbial metabolites. Short-chain fatty acids (SCFAs) are recognized as major microbial metabolites that serve as vital energy sources for colonic cells and exert regulatory effects on the host immune system. Lower SCFA production is associated with elevated levels of the genus *Anaerobacterium* [54]. We found that *Anaerobacterium* abundance was lower in DSS-treated mice after LIPUS treatment at an intensity of 0.5 ​W/cm^2^ (Fig. 6J). However, the intestinal microflora structure of mice treated with LIPUS at an intensity of 0.5 ​W/cm^2^ was significantly different from that of mice treated only with DSS (Fig. 6B).

Notably, abdominal LIPUS stimulation affected the inflammatory response in both the colon and the brain. However, the optimal intensities for modulating bacterial composition and inflammatory responses in the colon differed. The biochemical and histopathological changes in mice with DSS-induced colonic inflammation and neuroinflammation are intensity-dependent. The behavioral changes observed after LIPUS treatment likely result from interactions between gut inflammation, microbiome alterations, and vagal signaling. The anti-inflammatory properties of LIPUS may reduce gut pro-inflammatory cytokines, helping to restore homeostasis and dampen neuroinflammatory responses [55]. By reducing vagal activation, LIPUS may prevent excessive CNS inflammation, which could lead to behavioral improvements. Additionally, LIPUS-induced changes in the gut microbiota, possibly through metabolites like SCFAs, could further influence vagal signaling [56]. Zhou et al. examined the behavioral effects of DSS-induced colitis in mice, suggesting that the observed behavioral abnormalities in the DSS model are more likely associated with the core symptoms of ulcerative colitis rather than directly attributed to pain or psychological distress [57]. In our experiment, both the total distance traveled and movement in the central area during the OFT decreased in the DSS group, consistent with previous studies on DSS-induced colitis [[58], [59], [60]]. Weight reduction occurred in both the DSS and treatment groups; however, the treatment group showed increased central movement, indicating that behavioral differences were not due to weight loss. The DSS group's reduced willingness to explore the central area is linked to anxiety rather than pain. Therefore, the degree of weight loss does not impact our behavioral results, reinforcing that anxiety drives the reduced movement observed in the DSS group. A limitation of our study is that the LIPUS parameters used were the same as those in our previous study [22,23]. Further investigations are required to optimize ultrasound parameters to modulate intestinal microflora structure and lessen inflammatory responses, as well as to determine how abdominal LIPUS alters the gut microbiota and potentially contributes to the alleviation of the inflammatory responses in both the colon and the brain.

This study demonstrated that abdominal LIPUS effectively inhibited the inflammatory response in both the colon and the brain of mice. LIPUS treatment reduced gut barrier damage and lowered the serum LBP and LPS levels in DSS-treated mice. Consequently, LIPUS alleviated the symptoms of DSS-induced colitis and associated behavioral disorders. In addition, abdominal LIPUS altered the gut microbiota composition and fecal metabolite profile of DSS-treated mice. LIPUS stimulation could alleviate the fecal SCFA inhibition caused by the reduced *Anaerobacterium* abundance in DSS-treated mice. In neurodegenerative diseases, the targeting of the stages of intestinal inflammation for abdominal LIPUS stimulation may have the potential to alleviate or limit the progression of the diseases to the CNS and could improve the quality of life of patients and their families. Our findings suggest that the gut–brain axis might play a significant role in the protective effects of LIPUS against behavioral disorders and DSS-induced colitis symptoms.

## Author Contributions

Feng-Yi Yang and Yi-Ju Pan designed the study. Cong-Yong Gao, Yi-Ju Pan, Wei-Shen Su, Chun-Yi Wu and Ting-Yu Chang performed the animal study. Cong-Yong Gao and Wei-Shen Su helped performed statistical analysis and figure production. Feng-Yi Yang drew the manuscript. Feng-Yi Yang and Cong-Yong Gao revised the paper. Feng-Yi Yang and Yi-Ju Pan supplied fundings. All authors have read and agree to the published version of the manuscript.

## Data availability

The data that support the findings of this study are available on request from the corresponding author.

## Funding

This study was supported by grants from the 10.13039/100020595National Science and Technology Council of Taiwan (no. NSTC 113-2218-E-A49-031- and NSTC 111-2314-B-A49-045-MY3), the FEMH-NYCU Joint Research Program (no. 113DN22 and 112DN27).

## Declaration of competing interest

The authors declare that they have no known competing financial interests or personal relationships that could have appeared to influence the work reported in this paper.
