# Supplementary material for: Abdominal ultrasound stimulation alleviates DSS-induced colitis and behavioral disorders in mice by mediating the microbiota–gut–brain axis balance
Source: Neurotherapeutics. 2024 Nov 22;22(2):e00494. doi: 10.1016/j.neurot.2024.e00494 (PMC12014354; doi:10.1016/j.neurot.2024.e00494)
Supplement: Multimedia component 1 [file mmc1.docx]

**Tables**

Table S1. Disease activity index scoring.

| Score | Weight loss | Stool consistency | Bleeding |
| --- | --- | --- | --- |
| 0 | None | Normal stool | No bleeding |
| 1 | 1-5% | Slightly loose stool | Few blood-tinged stools |
| 2 | 5-10% | Loose stools | Slight bleeding |
| 3 | 10-15% | Watery stool | Gross bleeding |
| 4 | >15% | Severe diarrhoea | Blood filling the whole colon |

Table S2. Histological grading of colitis.

| Feature graded | Grade | Description |
| --- | --- | --- |
| Inflammation | 0 | None |
|  | 1 | Slight |
|  | 2 | Moderate |
|  | 3 | Severe |
| Extent | 0 | None |
|  | 1 | Mucosa |
|  | 2 | Mucosa and submucosa |
|  | 3 | Transmural |
| Regeneration | 4 | No tissue repair |
|  | 3 | Surface epithelium not intact |
|  | 2 | Regeneration with crypt depletion |
|  | 1 | Almost complete regeneration |
|  | 0 | Complete regeneration or normal tissue |
| Crypt damage | 0 | None |
|  | 1 | Basal 1/3 damaged |
|  | 2 | Basal 2/3 damaged |
|  | 3 | Only surface epithelium lost |
|  | 4 | Entire crypt and epithelium lost |
| Percent involvement | 1 | 1-25% |
|  | 2 | 26-50% |
|  | 3 | 51-75% |
|  | 4 | 76-100% |

**Figure**


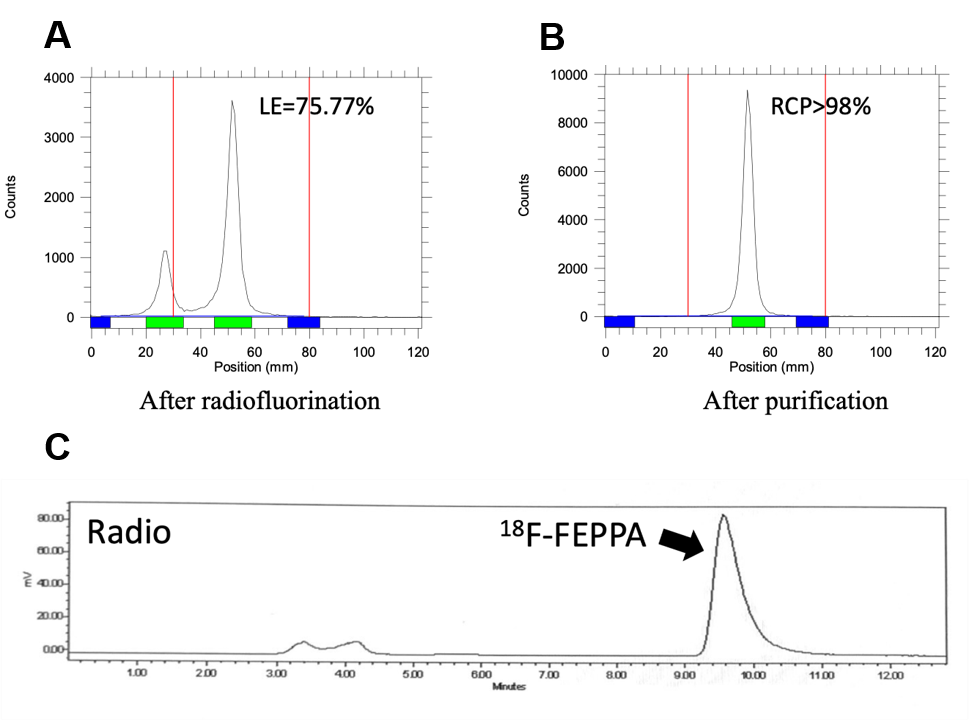


**Figure S1**. HPLC Quality Control Results of ^18^F-FEPPA. (A) Radiolabeling efficiency and (B) radiochemical purity of ^18^F-FEPPA. (C) The retention time of purified 18F-FEPPA was approximately 10 minutes. (LE: labeling efficiency; RCP: radiochemical purity)
