# Supplementary material for: Abdominal ultrasound stimulation alleviates DSS-induced colitis and behavioral disorders in mice by mediating the microbiota–gut–brain axis balance
Source: Neurotherapeutics. 2024 Nov 22;22(2):e00494. doi: 10.1016/j.neurot.2024.e00494 (PMC12014354; doi:10.1016/j.neurot.2024.e00494)

Blot images:

Fig.4 (D) ZO-1 (8% gel)

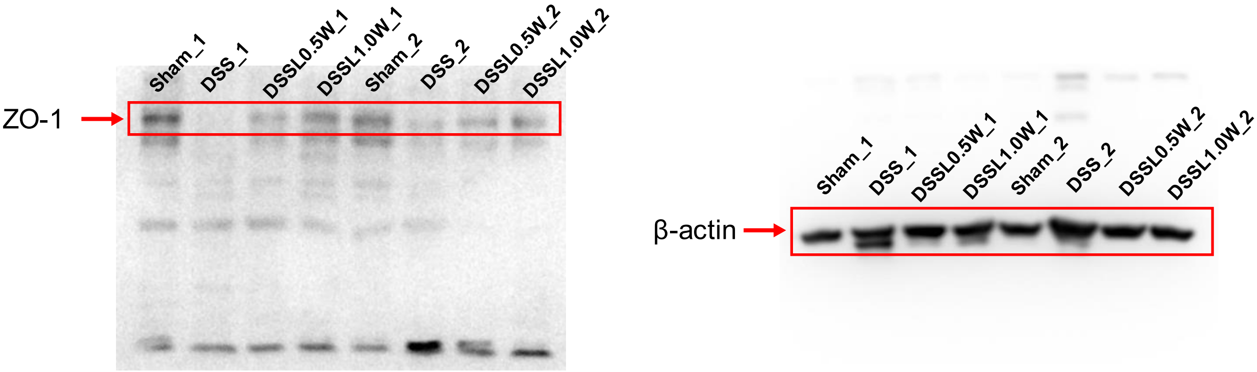

Fig.4 (D) ZO-1 (8% gel)

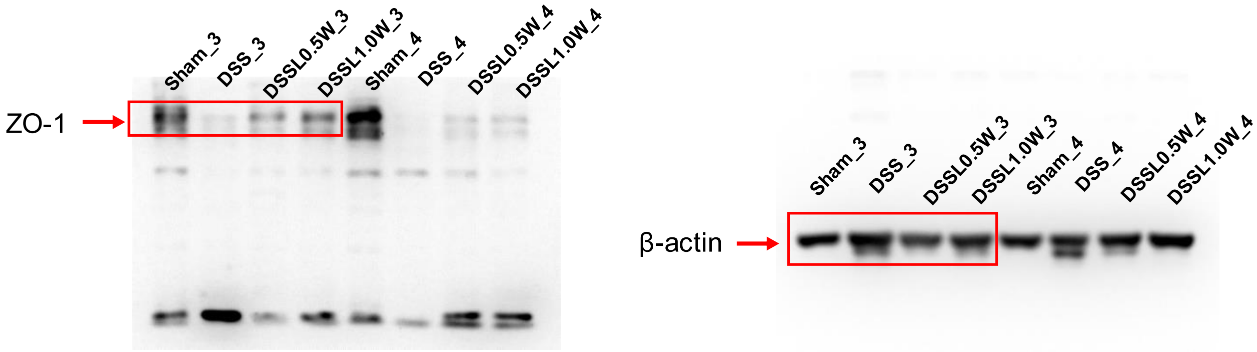

Fig. 4 (D) ZO-1 (8% gel)

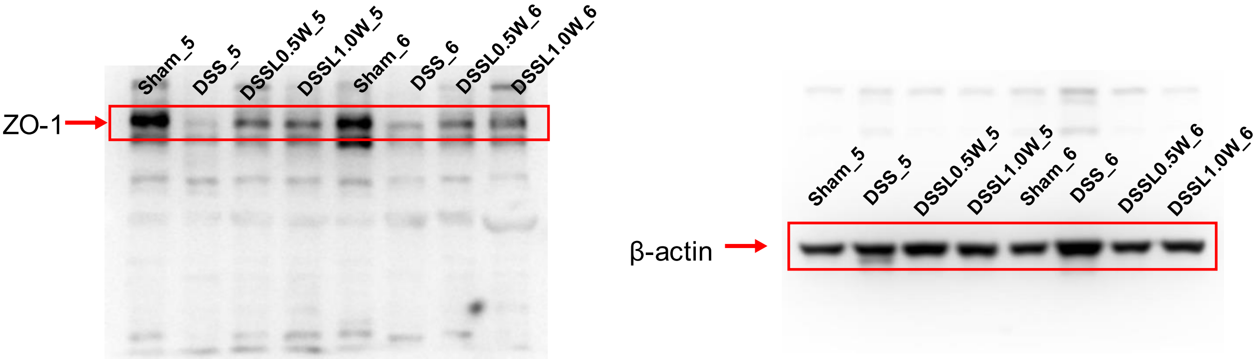

Fig.4 (D) ZO-1 with marker

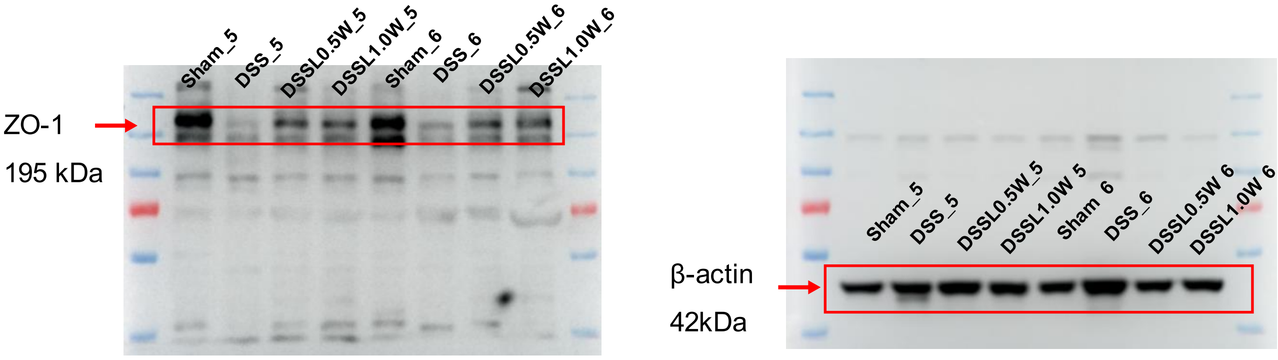

Blot images:

Fig.4 (E) Occludin (12% gel)

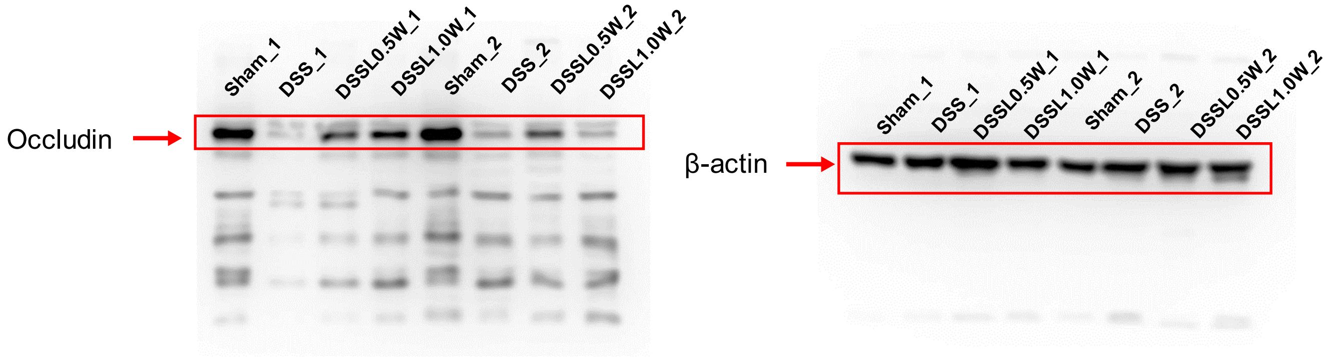

Fig.4 (E) Occludin (12% gel)

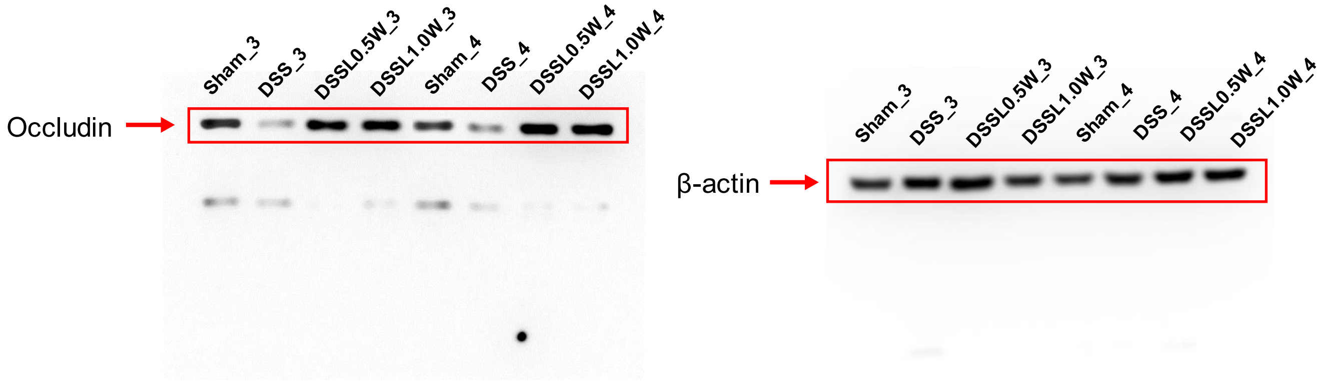

Fig.4 (D) Occludin (12% gel)

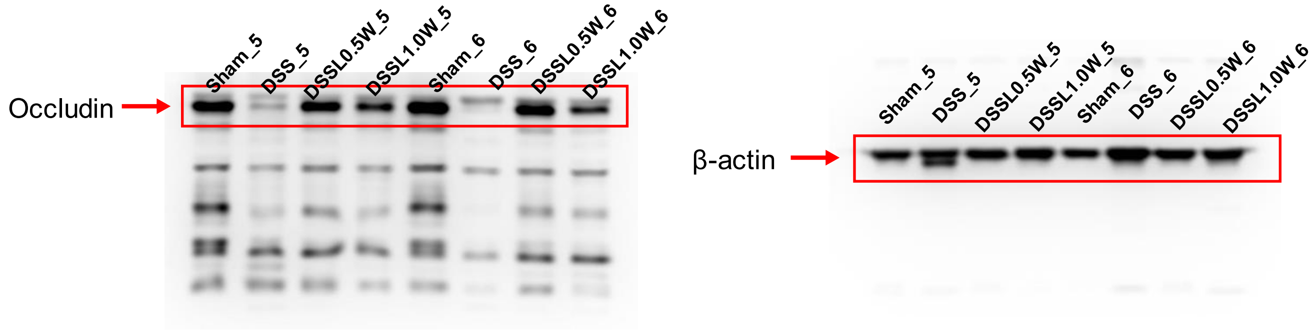

Fig.4 (D) Occludin with marker

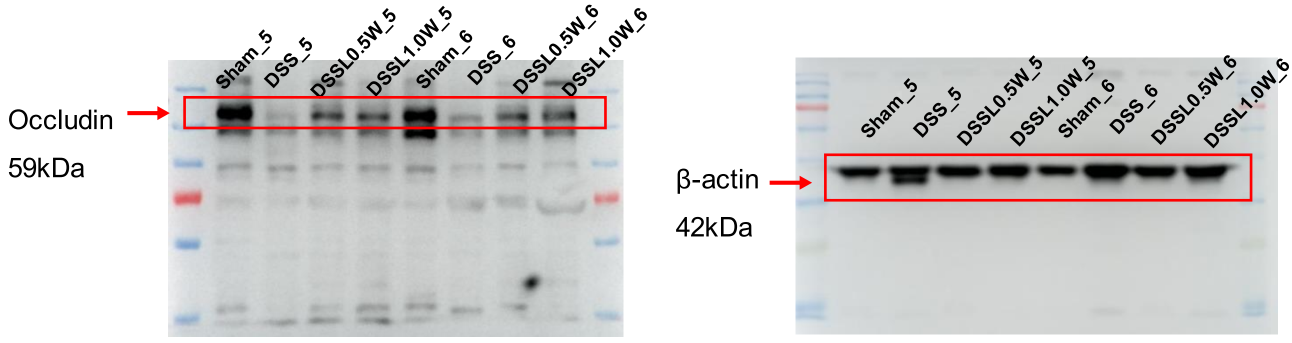

Blot images:

Fig.4 (F) iNOS (8% gel)

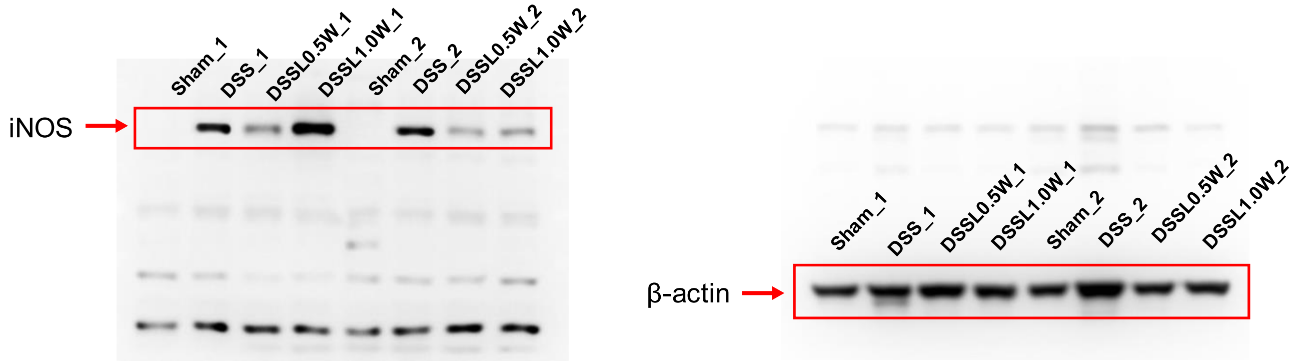

Fig.4 (F) iNOS (8% gel)

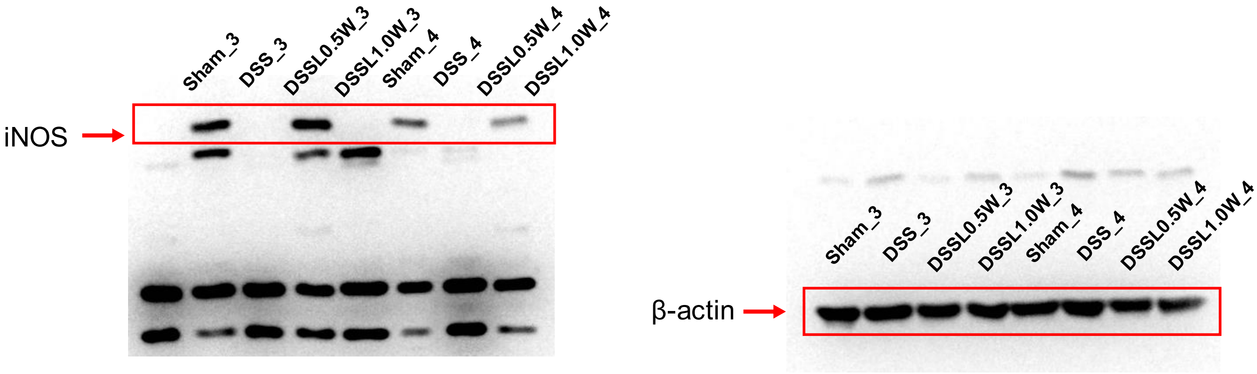

Fig.4 (F) iNOS (8% gel)

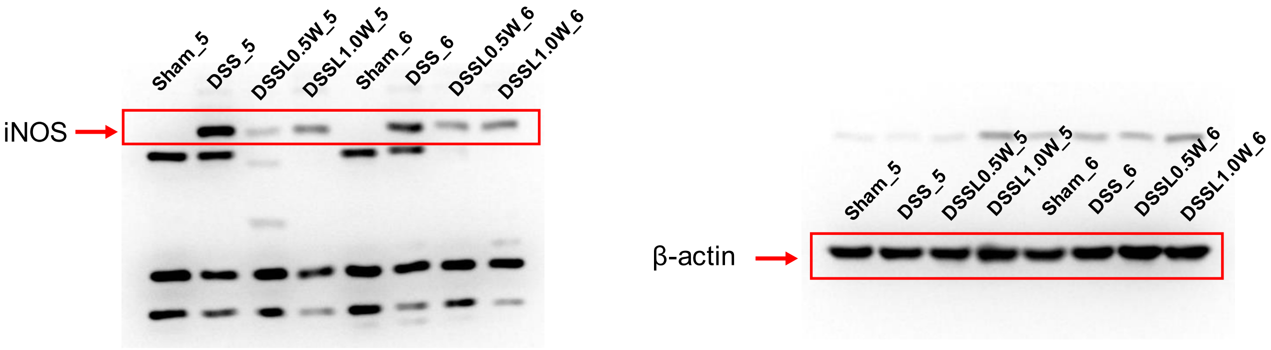

Fig.4 (F) iNOS with marker

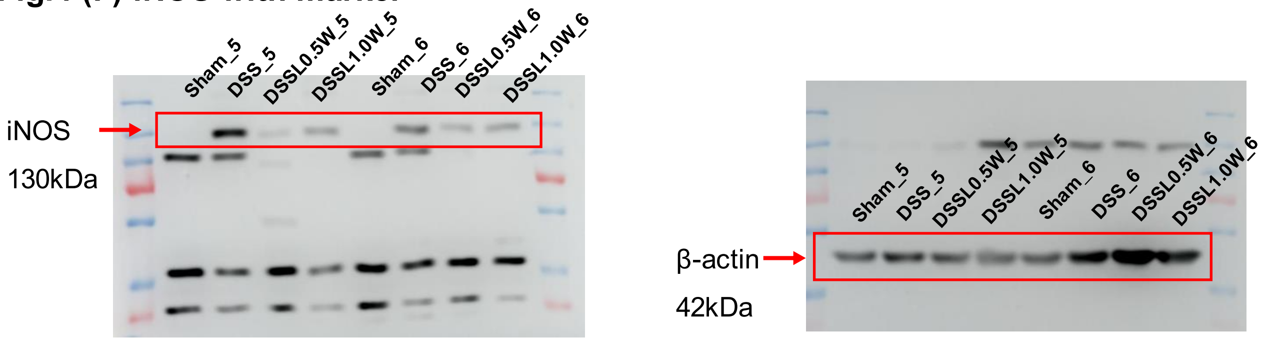

Supplement: Multimedia component 2 [file mmc2.pdf]
